# A scalable framework for UAV-based point cloud reconstruction and organ segmentation of field-grown cotton in complex field environments

**DOI:** 10.3389/fpls.2026.1854261

**Published:** 2026-06-23

**Authors:** Hao Qiu, Yunjie Zhao, Xiaoyan Meng, Yuxiang Wang, Xiaoyu Li, Haoyuan Niu, Liang Yu, Quanwen Mu, Sibo Meng, Shuai Yin

**Affiliations:** 1 College of Computer and Information Engineering, Xinjiang Agricultural University, Urumqi, China; 2 Ministry of Education Engineering Research Center for Intelligent Agriculture, Urumqi, China; 3 Xinjiang Agricultural Informatization Engineering, Technology Research Center, Urumqi, China; 4 College of Agriculture, Xinjiang Agricultural University, Urumqi, China

**Keywords:** 3D point cloud, field-grown cotton, neural radiance fields, semantic parsing, UAV-based 3D reconstruction

## Abstract

Accurate characterization of plant 3D architecture and semantic parsing of key cotton organs represent an essential prerequisite for crop phenotyping, precision field management, and cultivar breeding and selection. However, the overall 3D structure of field-grown cotton plants is highly complex in field environments. The morphological traits and spatial distribution of various organs are modulated by multiple factors including genetics, environmental conditions, and cultivation management practices, resulting in pronounced phenotypic variation under field conditions. Thus, this study proposed an integrated technical framework for UAV-based 3D reconstruction and organ semantic segmentation tailored for field-grown cotton. High-fidelity 3D point clouds of the crop canopy are generated by coupling an field-adapted low-altitude unmanned aerial vehicle (UAV) acquisition strategy with neural radiance fields (NeRF). Despite these attractive characteristics, segmenting and analyzing such intricate point clouds can be quite challenging. To effectively parse these complex geometric structures, a novel deep learning architecture named FieldCotSeg-Net is introduced. This model integrates an Anisotropy-aware Local Attention (ALA) module and a Hierarchical Feature Refinement Gate (HFRG) module to capture fine-grained features for precise point cloud segmentation. Experimental results demonstrate that the proposed model achieves outstanding performance on both the Huaxing3Dcot and Crops3D cotton datasets. On the Huaxing3Dcot dataset, the model yields a mean intersection over union (mIoU) of 75.7%, representing a 4.5% improvement over the baseline model. On the Crops3D cotton dataset, after retraining the model on this dataset, it attains an mIoU of 74.5%, showing substantial adaptability and effectiveness in organ segmentation. This technical system provides a scalable and robust solution for field cotton phenotyping analysis in breeding research and commercial production.

## 1 Introduction

Cotton is a globally significant strategic crop with high economic value (Liu et al., 2025c), relies heavily on accurate, high-throughput plant phenotypic data to enhance yield, improve quality, and optimize cultivation management. Traditional manual field measurement of cotton phenotypic information is not only time-consuming, labor-intensive, and costly, but also prone to systematic human errors and may cause irreversible damage to plant morphology, rendering it entirely inadequate for the high-throughput phenotyping demands of modern cotton breeding and large-scale cultivation (Li et al., 2025b). The rapid development of UAV remote sensing technology has effectively broken through the inherent limitations of conventional phenotypic data collection. Its advantages of flexible operation, high acquisition efficiency, and non-destructive workflow have made it a mainstream technique for acquiring crop phenotypic information at the field scale (Xiao et al., 2024). Multi-view sequential images captured by low-altitude UAVs, combined with 3D reconstruction techniques, can non-destructively reconstruct the complete 3D geometric morphology of cotton plants, generating high-precision 3D point cloud datasets. These datasets lay the critical data foundation for organ-level fine segmentation and quantitative extraction of phenotypic parameters (Shi et al., 2022).

Although UAV-based 3D reconstruction has opened new avenues for crop phenotyping research, constructing high-quality point cloud datasets and achieving organ-level semantic segmentation in complex field environments still face two major challenges. The first challenge pertains to data acquisition and 3D reconstruction. Current approaches for acquiring crop 3D point cloud data are mainly divided into two categories: active sensing and passive image-based reconstruction (Yu et al., 2024). Active sensing, relying on devices such as LiDAR that emit detection signals for 3D perception, offers high single-point measurement accuracy but suffers from high equipment and operational costs and relatively sparse data collection in large-scale field scenarios (Tang et al., 2024; Omia et al., 2026). In contrast, passive image-based reconstruction using low-altitude UAVs combines advantages such as high operational efficiency, wide field coverage, and low equipment investment, making it highly compatible with large-scale cotton cultivation scenarios (Liu et al., 2021; Gao et al., 2022). However, traditional multi-view stereo (MVS) methods (Meinen and Robinson, 2020) exhibit insufficient reconstruction accuracy and prominent point cloud noise and redundancy under complex field conditions such as fluctuating illumination and severe organ occlusion (Sankaramaddi et al., 2026; Zhai et al., 2024; Liu et al., 2025b; Li et al., 2023, 2025a. In recent years, the emergence of Neural Radiance Fields (NeRF) (Mildenhall et al., 2021) has provided a new paradigm to address this dilemma, enabling the generation of more continuous and complete 3D structures from sparse images. Therefore, integrating NeRF with UAV acquisition strategies to construct high-fidelity 3D point cloud datasets of field-grown cotton is a primary issue that needs to be resolved.

The second challenge focuses on organ-level semantic segmentation methods for 3D point clouds. Accurately achieving organ-level semantic segmentation of cotton plant point clouds is a prerequisite for the quantitative extraction of core architectural phenotypic parameters such as plant height, fruit branch number, leaf area, and stem diameter. Moreover, it is a necessary foundation for dissecting the genetic mechanisms of cotton architecture, molecular breeding for ideotypes, and intelligent variable-rate management in the field (Zhu et al., 2024a; Liu et al., 2025a; Jiang et al., 2025). Nevertheless, the 3D structure of field-grown cotton plants is highly complex, with the morphological characteristics and spatial distribution of each organ jointly regulated by genetic, environmental, cultivation management, and other factors, leading to significant phenotypic variability. Traditional segmentation methods based on geometric rules or hand-crafted features struggle to adapt to this diversity (Yang et al., 2025; Liu et al., 2025d; Fatima et al., 2025; Bryson et al., 2024).

In recent years, deep learning-based point cloud segmentation networks (e.g., PointNet++ (Qi et al., 2017), PointMLP (Ma et al., 2022) have achieved significant progress on general-purpose point cloud benchmarks (Tan et al., 2025; Peng et al., 2023). For crop point cloud segmentation, extensive algorithm optimization efforts have been reported, and these methods have demonstrated excellent performance in single-plant segmentation tasks under controlled indoor environments. For instance, Zhu et al. (2024b) achieved high-precision automated phenotypic parameter calculation using indoor potted plant point clouds. Shen et al. (2024) performed accurate organ segmentation and multi-dimensional phenotypic feature extraction on point clouds of two types of cotton seedlings grown indoors. However, these methods are primarily designed for crop point clouds from idealized indoor conditions. When directly applied to field-grown crop point clouds, which are more complex, they often fail to capture anisotropic local structural features and exhibit insufficient discriminative power for fine-grained organ boundaries. Hence, there is an urgent need to design deep learning architectures tailored to the characteristics of field-grown cotton point clouds to address the challenge of accurately segmenting multiple morphotypes and varieties of cotton organs under complex environmental and occlusion conditions.

Addressing the two major challenges above, this study constructs a technical framework for 3D point cloud reconstruction and organ-level semantic segmentation of field-grown cotton, aiming to provide a highly robust and scalable technical solution for high-throughput phenotyping of field cotton.

1. We adopt a UAV-integrated NeRF-based 3D point cloud reconstruction method for field cotton. By establishing a standardized field image acquisition protocol using low-altitude UAVs and combining it with Neural Radiance Fields, we achieved high-precision 3D point cloud reconstruction of cotton plants in complex field scenes and constructed a dedicated dataset named Huaxing3Dcot, providing high-quality data support for subsequent organ-level semantic segmentation.
2. We propose a novel deep learning model, FieldCotSeg-Net. For point cloud semantic segmentation, we design an Anisotropy-aware Local Attention (ALA) module to adaptively capture the anisotropic geometric features of different cotton organs, thereby enhancing the model’s robustness to field phenotypic variability. Meanwhile, a Hierarchical Feature Refinement Gate (HFRG) module is constructed to effectively filter redundant noise during feature propagation, achieving efficient fusion of shallow geometric details and deep semantic features and improving segmentation accuracy.
3. Systematic experimental validation was conducted on our self-constructed Huaxing3Dcot cotton dataset and the public Crops3D cotton dataset (Zhu et al., 2024a). The results demonstrate that the proposed model achieves a mean Intersection over Union (mIoU) of 75.7% on the Huaxing3Dcot dataset, outperforming the baseline model by 4.5%. On the Crops3D dataset, after retraining the model on this dataset, it achieves an mIoU of 74.5%, showing significant improvements in cross-dataset generalization performance and organ segmentation effectiveness.

## 2 Methods

### 2.1 3D reconstruction pipeline for field-grown cotton

#### 2.1.1 Data acquisition area

**Figure 1:**
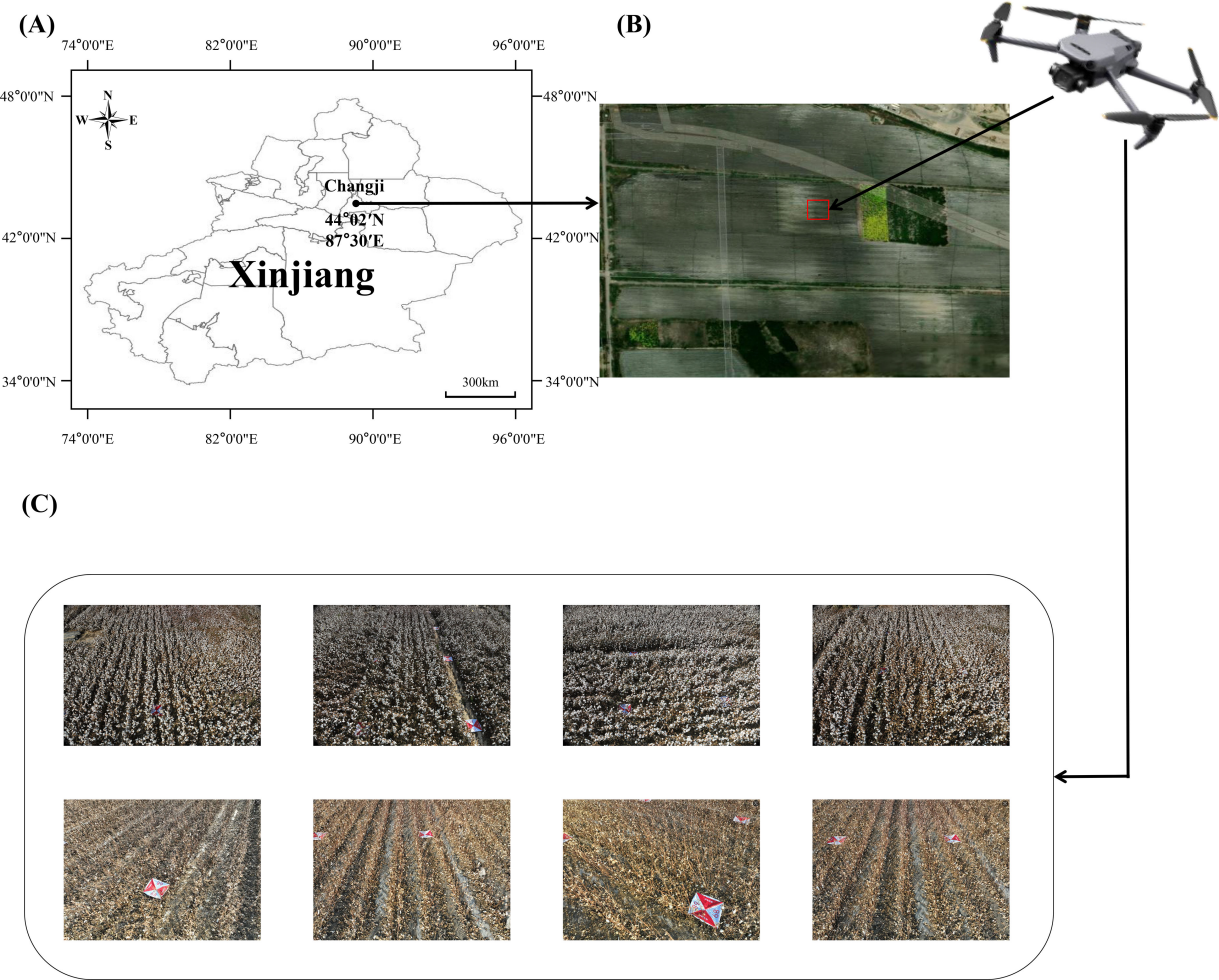
Overview of image data acquisition for field-grown cotton. **(A)** Schematic diagram of the geographic location of the sampling base. **(B)** Real scene of the field experiment acquisition area. **(C)** Multi-view images of field-grown cotton acquired by UAV at two distinct growth stages.

The data acquisition site was located at Huaxing Farm in Changji City, Xinjiang Uygur Autonomous Region, China (44^°^02^′^ N, 87^°^30^′^), as depicted in **Figure 1**. The site is a large-scale field planting area for cash crops. The sampling plot covered an area of 5.5 m × 5.5 m and comprised six standardized cotton rows. Within this plot, the row spacing was maintained at 0.6 m, and the intra-row plant spacing was set to 0.1 m.

Based on the above sampling plot, data collection was performed at two key stages: before harvest (October 25, 2025) and after the first round of mechanical picking (November 2, 2025). Weather conditions were identical on both days (clear skies), which effectively eliminated interference from weather variability on data quality. This study simultaneously collected UAV-based aerial RGB images and refined field ground-truth phenotyping measurements. A total of 516 RGB aerial images were acquired, including 254 images taken before harvest and 262 images taken after the first round of mechanical picking. All data collection for each stage was completed in a single day to ensure the timeliness and consistency of the dataset.

#### 2.1.2 UAV data acquisition flight path planning and design

In this study, a DJI Mavic 3M UAV (DJI, Shenzhen, China) was employed for field image acquisition of field-grown cotton. The flight mission was configured with core operational parameters including a relative altitude of 5 m and a flight velocity of 0.5 m/s. All acquired images were uniformly saved in JPG format, with a single-frame resolution of 5280×3956 pixels. Each image was attached with complete EXIF (version 0230) metadata, including GPS positioning, flight attitude, camera parameters, and absolute flight height, providing comprehensive data support for high-precision multi-view 3D reconstruction of cotton plants.

**Figure 2:**
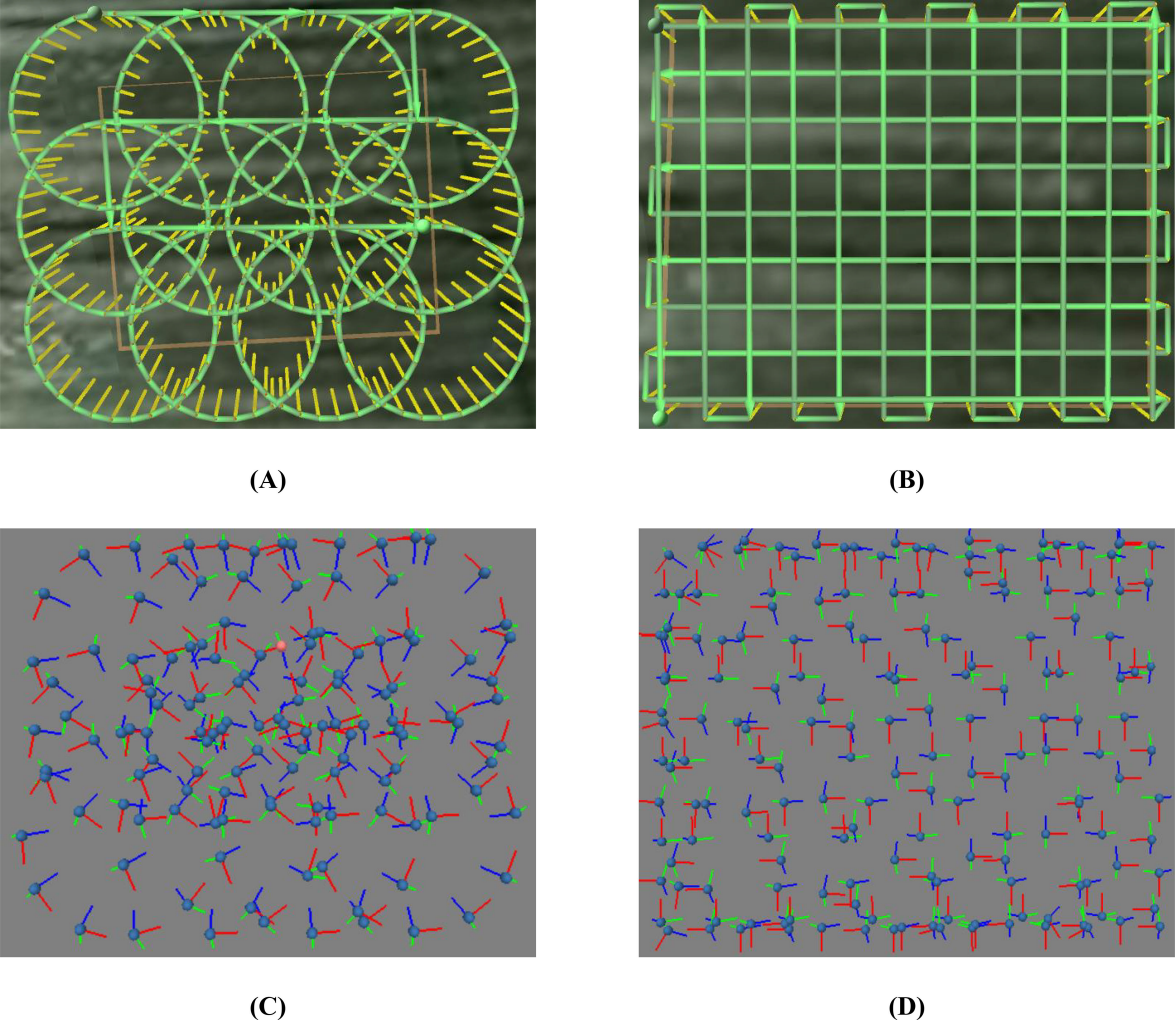
Flight path planning and design for UAV acquisition. **(A)** Flight trajectory of the CCO path. **(B)** Flight trajectory of the grid path. **(C)** Distribution of image capture points for the CCO path. **(D)** Distribution of image capture points for the grid path.

This study specifically designed hybrid acquisition schemes: the Cross Circular Oblique (CCO) (Xiao et al., 2024) path and the grid path, with the path layout shown in **Figure 2**. The CCO path can penetrate the leaf occlusion on the upper cotton canopy and fully capture images of subtle organs in the middle and lower plant parts, such as cotton bolls, stems, and leaves. The grid path compensates for the acquisition blind spots of the CCO path on the lateral sides of plants and between field rows. For parameter configuration, the CCO path utilized a 45°camera pitch angle, a circular overlap of 45%, an acquisition rate of 12 frames per circle, and a circular radius of 2.5 m. The grid path maintained the same 45°camera angle, with both forward and side overlaps set strictly at 45% and a capture interval of 2.5 s, ensuring automatic temporal continuity and precise spatial registration. The collaborative operation of the two paths effectively overcomes multiple technical drawbacks in the single acquisition mode, including severe canopy occlusion and missing lateral-view data.

#### 2.1.3 UAV data processing

All UAV flight operations were equipped with a DJI real-time kinematic (RTK) base station to ensure precise positioning of the UAV during data acquisition. In addition, ground control points (GCPs) were deployed at the four corner points of the acquisition area as the geographic reference for photogrammetric processing, thereby improving the spatial accuracy and scale consistency of orthophotos and 3D point cloud models. The exact coordinates of these GCPs were acquired using a Qianxun Spatial SR3 Pro RTK survey instrument (QX Spatial Information Network Co., Ltd., Shanghai, China), a high-precision Beidou receiver integrated with the QX FindCM (5 constellations, 16 frequencies) service, featuring stable measurement performance and excellent accuracy.

The acquired images were imported into Agisoft Metashape Professional software (Version 2.2.2) (Agisoft, 2020) for full-process photogrammetric processing. The software performed image mosaicking and registration based on the Structure from Motion (SfM) algorithm, constructed a 3D sparse point cloud model of field-grown cotton, and exported camera pose information to provide preliminary data for subsequent dense point cloud reconstruction. The entire reconstruction process was referenced to GCPs, which serve multiple functions, including correcting geographic coordinates, assisting in high-precision image mosaicking and matching, and acting as a scale reference to ensure the spatial dimensional accuracy of the reconstructed model. In this study, GCP markers were manually identified and labeled in the image subsets corresponding to each plot, and the measured coordinate data were imported and associated to ensure the accuracy of the modeling and registration workflows.

#### 2.1.4 Dense point cloud reconstruction of cotton fields

After image alignment was completed in Agisoft, the estimated camera poses of each plot image set were exported for training the NeRFacto model in NeRF Studio software (Tancik et al., 2023). The original images and corresponding camera poses were then converted to the format compatible with NeRF Studio to construct a standardized training dataset. The core objective of this study was to achieve high-precision 3D reconstruction of field-grown cotton plants, with reference to the findings of Arshad et al. (2024) and James et al. (2025).The former systematically evaluated several mainstream NeRF frameworks for multi-scale 3D reconstruction of maize from RGB images in field scenes, confirming that NeRFacto achieves superior accuracy and robustness over frameworks such as Instant-NGP and TensorRF. The latter applied NeRFacto to sorghum canopies, providing a practical pipeline that we referenced and adapted as a methodological template for the 3D reconstruction of field cotton.

**Figure 3:**
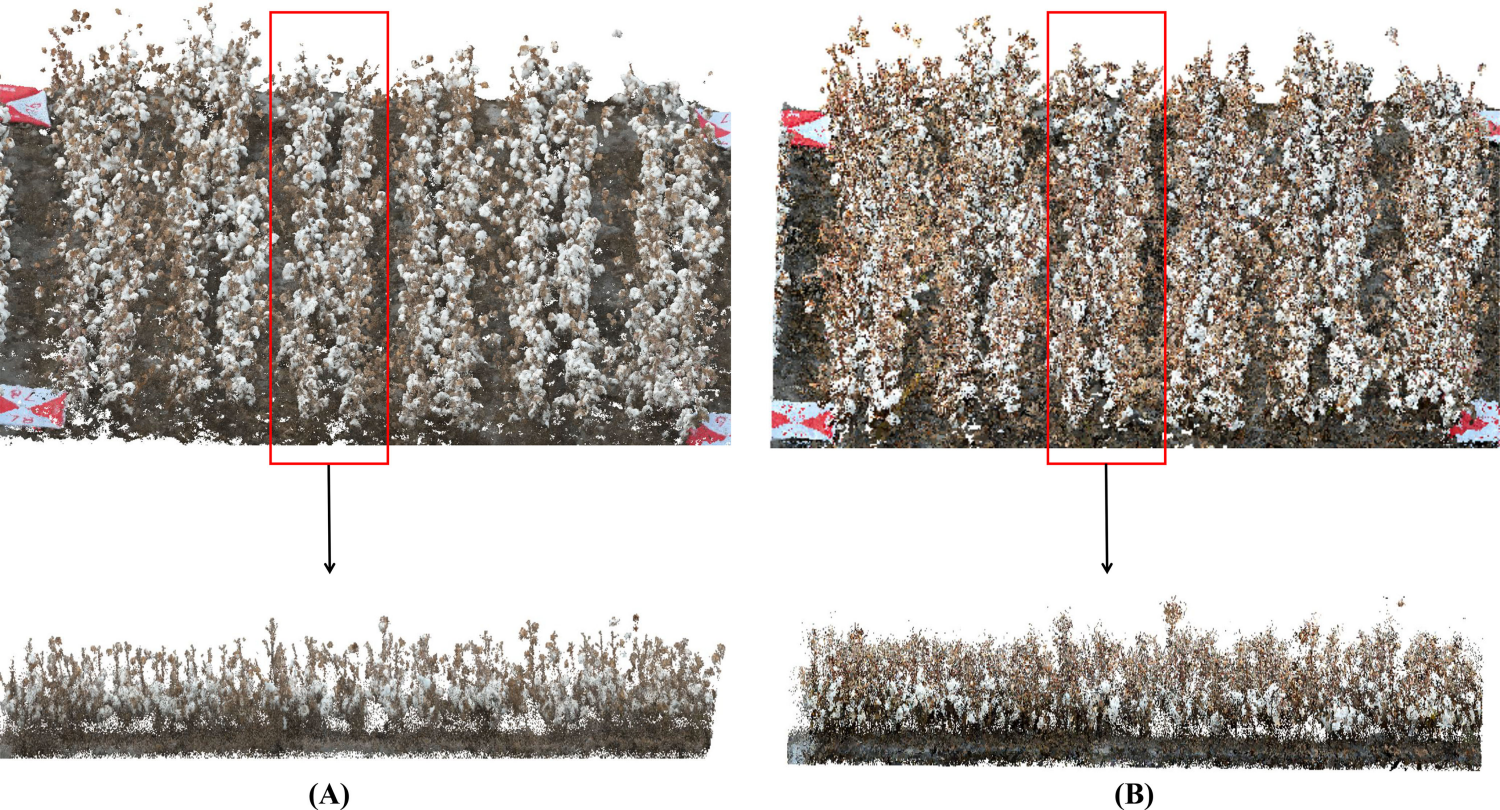
Visual comparison of field-grown cotton point cloud performance reconstructed by different models. **(A)** Reconstructed point cloud result of the NeRFacto model. **(B)** Reconstructed point cloud result of the native 3DGS model.

To further select the optimal scheme suitable for cotton reconstruction, this study comparatively tested the reconstruction performance of the native 3DGS model (Kerbl et al., 2023) and NeRFacto. To guarantee experimental fairness, both algorithms were strictly initialized with the identical set of input images and SfM-derived camera parameters, and both were configured to output a unified point cloud of exactly 15 million points. The visual comparison results of the point clouds from the two models are shown in **Figure 3**. As can be seen from the figure, NeRFacto reconstructs point clouds with colors that more closely match real field cotton, exhibits fewer noise points, and achieves significantly better reconstruction performance in the root region compared to the native 3DGS model. Based on the evaluation of reconstruction accuracy, NeRFacto was ultimately designated as the optimal solution for generating high-precision cotton point clouds.

#### 2.1.5 Construction of the Huaxing3Dcot dataset

**Figure 4:**
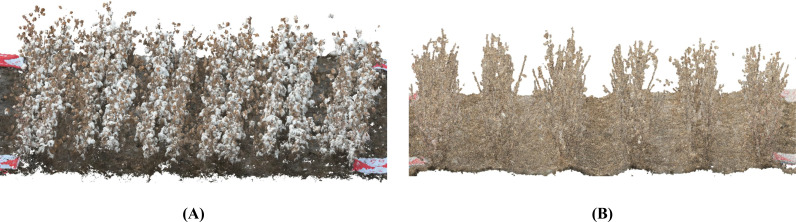
Point clouds of field-grown cotton at different growth stages. **(A)** Before harvest. **(B)** After the first mechanical harvesting.

To prioritize spatial precision over computational efficiency, we selected the NeRFacto-huge, the variant with the strongest reconstruction performance in the NeRFacto family, for high-precision 3D point cloud reconstruction. This model fully leverages its strengths in detail restoration and accuracy performance, effectively preserving the 3D structural characteristics of cotton plant canopies, stems, and bolls. The overall point cloud effect of the final reconstructed field-grown cotton is displayed in **Figure 4**.

**Figure 5:**
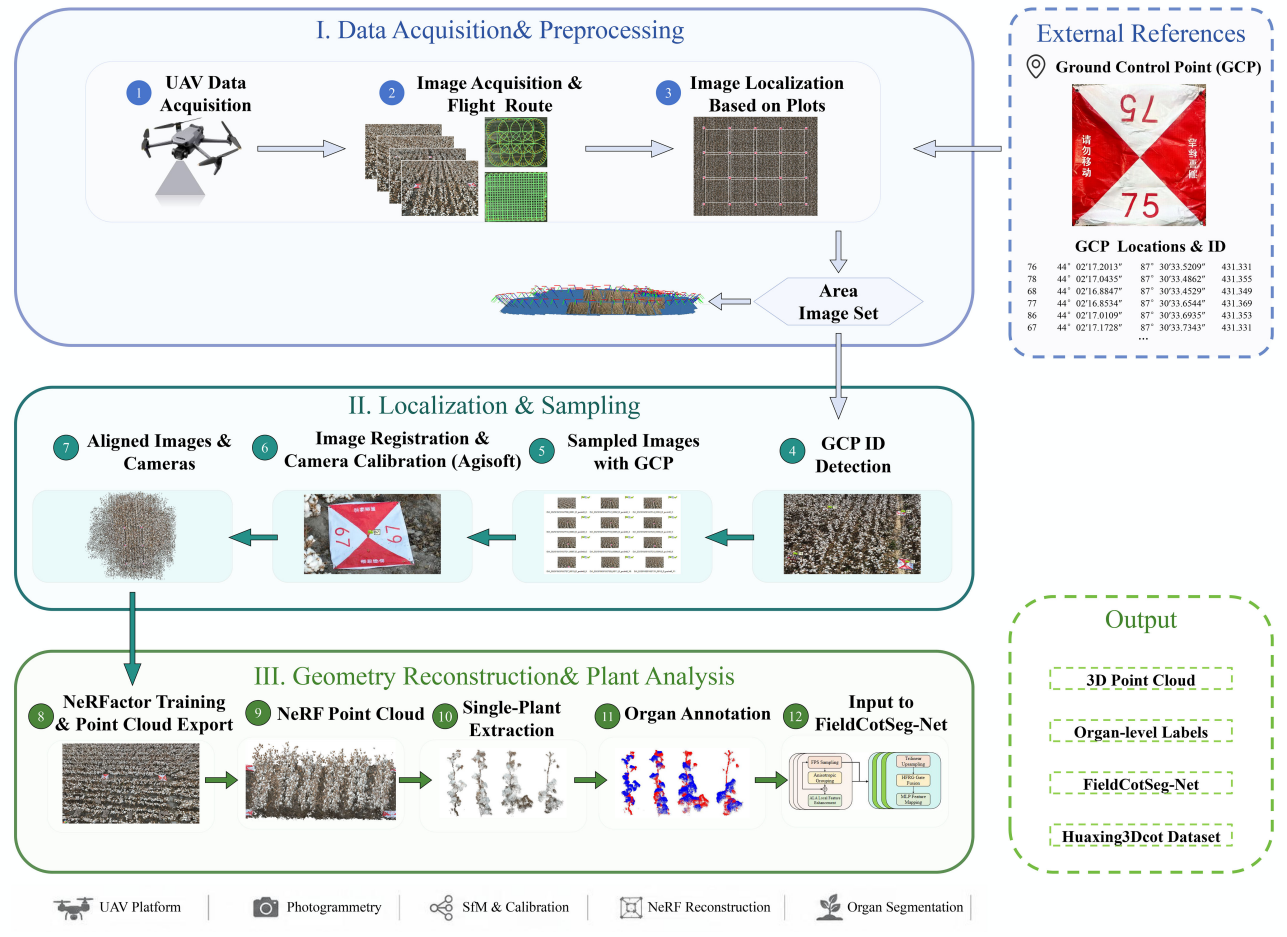
Construction workflow of the Huaxing3Dcot dataset from image acquisition to model input.

Following the completion of global point cloud reconstruction, refined post-processing was carried out using CloudCompare (Version 2.13.2) software. Accurate segmentation and extraction of individual cotton plant point clouds in the field were achieved via the 3D interactive functions of the platform, and fine semantic annotation of plant structures was completed using built-in professional tools. The overall standardized construction workflow of the Huaxing3Dcot dataset, covering the full link from image acquisition and 3D reconstruction to fine point cloud annotation and finally to model input, is illustrated in **Figure 5**.

**Figure 6:**
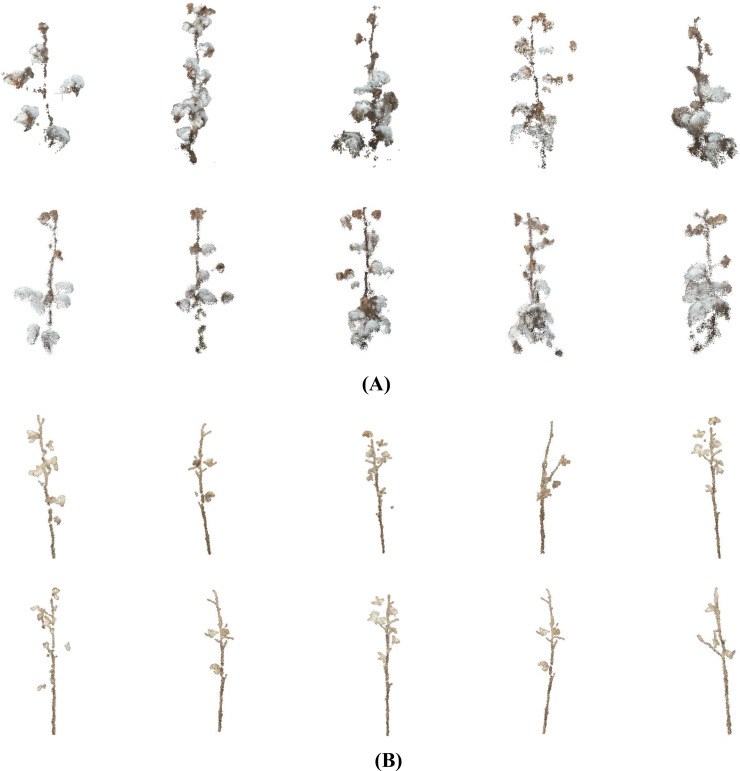
Visualization of sample point clouds from the Huaxing3Dcot dataset. **(A)** Individual cotton plant point cloud before harvest. **(B)** Individual cotton plant point cloud after the first mechanical harvesting.

Ultimately, valid individual cotton plant point cloud samples were integrated to construct a standardized, high-quality annotated dataset termed Huaxing3Dcot, which comprises 240 finely annotated individual cotton plants. Visualization results of partial individual plant point clouds from this dataset are presented in **Figure 6**.

**Figure 7:**
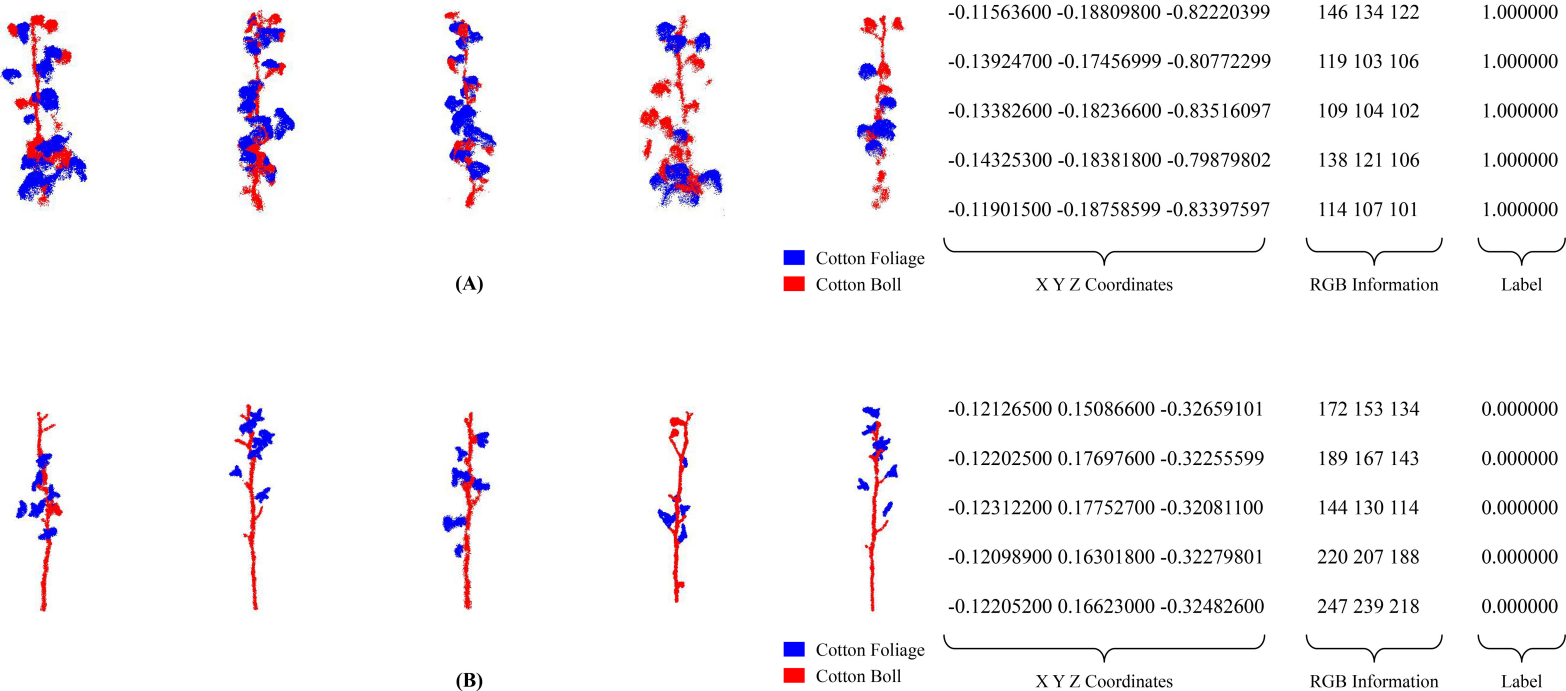
Annotated samples of the Huaxing3Dcot dataset. **(A)** Cotton point cloud sample before harvest. **(B)** Cotton point cloud sample after the first mechanical harvest.

Based on the established Huaxing3Dcot dataset, comparative experiments and module ablation verification were first performed in this study. The 240 field-grown cotton point cloud samples are divided into two subsets, including 55 samples collected on the eve of harvest and 185 samples obtained after the first mechanical harvest. Visualization results of typical annotated samples from the dataset are displayed in **Figure 7**.

### 2.2 FieldCotSeg-Net

**Figure 8:**
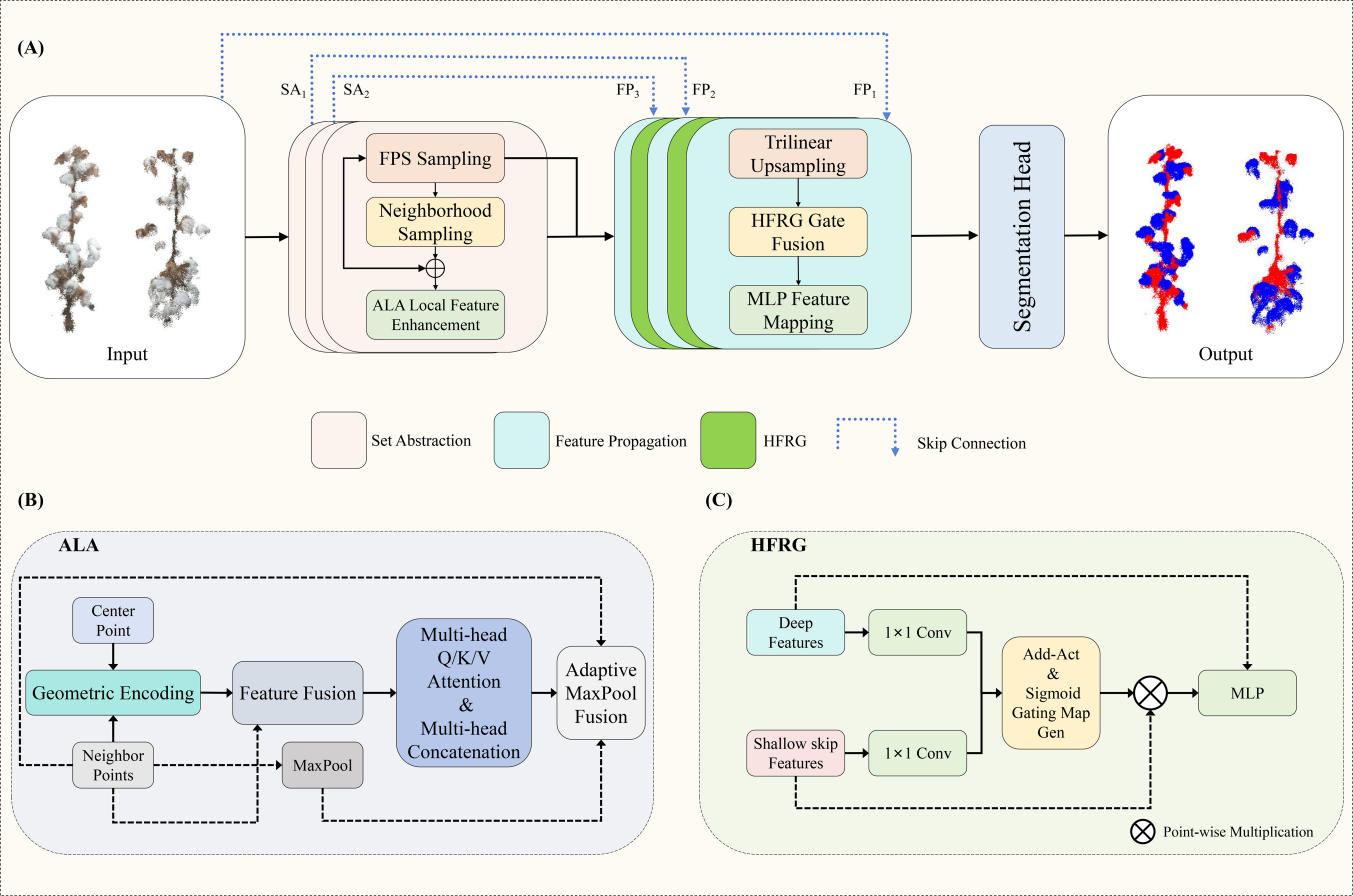
Schematic diagram of the overall network and core module structures. **(A)** Overall architecture of the FieldCotSeg-Net segmentation network. **(B)** Structure of the ALA module. **(C)** Structure of the HFRG module.

The cotton point cloud organ segmentation network FieldCotSeg-Net proposed in this study is built on PointNet++ with an encoder–decoder architecture. The network is structured into a multi-scale Set Abstraction encoder, a Feature Propagation decoder, and a point-wise segmentation head, as illustrated in **Figure 8**. The encoder adopts a multi-scale message aggregation strategy and conducts hierarchical downsampling and feature extraction via three set abstraction (SA) layers: SA1 and SA2 sample local centers through farthest point sampling, extract multi-scale local geometric features, and integrate the proposed Anisotropy-aware Local Attention (ALA) module instead of max-pooling to achieve adaptive feature aggregation for cotton organs’ anisotropic geometries, while SA3 aggregates global features to supply high-dimensional semantic information for decoding. The decoder contains three feature propagation (FP) layers that upsample deep features to the original resolution through k-nearest neighbor interpolation, and a Hierarchical Feature Refinement Gate (HFRG) module is deployed at FP2 and FP1 to filter shallow skip-connection features using deep semantic signals, reducing semantic confusion and enhancing boundary segmentation accuracy between cotton bolls and branches. The segmentation head uses two 1D convolutional layers to map point-wise features and output label probability distributions for two cotton organ categories, generating the final precise segmentation predictions.

#### 2.2.1 ALA module

The existing local aggregation operators in point cloud networks, such as max pooling, treat all points within a neighborhood equally and overlook the spatial variability of local geometric structures. However, the two types of organs on a cotton plant exhibit distinctly different local geometric characteristics. A branch extends linearly along its growth direction, and its local neighborhood shows a pronounced anisotropic distribution, with points densely distributed along the branch direction but sparsely in the perpendicular direction. In contrast, a cotton boll presents a nearly spherical closed surface, and its neighborhood distribution tends to be isotropic. A single scalar importance score cannot effectively capture the feature differences between these two structures. To address this issue, this paper proposes an Anisotropic-aware Local Attention (ALA) module. This module achieves adaptive aggregation of local features through a multi-head self-attention mechanism fused with geometric positional encoding. It should be noted that the term “anisotropy-aware” in this module does not refer to modifying the way the neighborhood is constructed. Instead, it means that direction-sensitive geometric positional encoding explicitly injects the spatial directional distribution information of points within the local neighborhood into the feature representation, enabling the subsequent attention mechanism to perceive directional distribution differences when computing aggregation weights. In this way, the module achieves adaptive representation of anisotropic local structures at the feature level.

The input of the ALA module consists of the central point **p***_i_* in a local region and its set of *K* neighboring points ${\left\{p_{j}\right\}}_{j=1}^{K}$, with corresponding point features ${\left\{f_{j}\right\}}_{j=1}^{K}$. To enable the model to perceive geometric distribution differences in the local neighborhood, a geometric descriptor is first constructed for each neighboring point. Specifically, the relative coordinates of each neighbor with respect to the central point are computed, and normalized relative distance is introduced to eliminate the influence of global scale variations of the point cloud. These two components are concatenated to form a 4-dimensional geometric descriptor, which is then mapped via a two-layer MLP to a geometric positional encoding $e_{ij}$ with the same dimension *C* as the feature dimension, as shown in Equation 1.

(1) $$g_{ij}=[p_{j}-p_{i},\;\frac{∥p_{j}-p_{i}{∥}_{2}}{\underset{k}{\max}∥p_{k}-p_{i}{∥}_{2}+\in }]\in {\mathbb{R}}^{4},\;e_{ij}={\text{MLP}}_{\text{pos}}(g_{ij})\in {\mathbb{R}}^{C}$$

where *∈* is a numerical stability term to avoid division by zero. The three-dimensional relative coordinate component $p_{j}-p_{i}$ in the geometric descriptor $g_{ij}$ retains complete directional information of the neighboring point relative to the central point, serving as the core of the anisotropy-aware capability of this module. For a branch structure that extends linearly along a certain direction, the relative coordinates of its *K* neighboring points are concentrated near that directional axis. for a nearly spherical cotton boll, the relative coordinates tend to be uniformly distributed across all directions. Mapping the above direction-sensitive geometric descriptor to a positional encoding via a multilayer perceptron and incorporating it into the subsequent computation of attention weights enables the model to generate differentiated responses to the directional distribution of neighboring points when aggregating local features, thereby explicitly introducing awareness of local anisotropic structures at the feature level. The introduction of normalized distance endows the positional encoding with scale invariance, which is particularly important for cotton point clouds—point cloud densities of different samples collected in the field vary, and the normalization operation ensures that the model remains robust to changes in sampling density. Finally, the geometric positional encoding is additively fused with the neighboring point features to obtain the geometry-enhanced feature ${\tilde{f}}_{j}=f_{j}+e_{ij}$.

On this basis, the ALA module uses a multi-head self-attention mechanism to aggregate local features. The mean of all geometrically enhanced features in the neighborhood is taken as the Query vector, and the geometrically enhanced features of each neighboring point are separately used as Key and Value vectors. After projection via the linear projection matrices **W***_Q_*, **W***_K_*, and **W***_V_*, the attention weights are computed independently within each attention head *h*, as shown in Equation 2.

(2) $$\alpha_{ij}^{h}=\frac{\exp\;(q^{h}\cdot {\left(k_{j}^{h}\right)}^{⊤}/\sqrt{d_{h}})}{\sum_{k=1}^{K}\exp\;(q^{h}\cdot {\left(k_{k}^{h}\right)}^{⊤}/\sqrt{d_{h}})}$$

Among them, *H* is the number of attention heads, *d_h_* = *C/H* is the feature dimension of each head, and $\sqrt{d_{h}}$ is the scaling factor used to prevent excessively large inner products from causing vanishing gradients. Since both the Key and Value vectors are projected from the feature ${\tilde{f}}_{j}$, which incorporates directional-sensitive geometric position encoding, the attention weight $\alpha_{ij}^{h}$ naturally embeds the perception of the spatial direction distribution of neighboring points during computation. The multi-head mechanism allows the model to independently perceive diverse local geometric relationships in different projection subspaces, thereby enabling the structural capability to cooperatively express the distinct geometric characteristics of two organ types – branches and bolls – within a single module. The choice of the number of attention heads *H* requires a trade-off between representational capacity and the feature capacity per head. Too few heads limit the geometric perception capability of each subspace, whereas too many heads result in an excessively small feature dimension *d_h_* = *C/H* per head, reducing the representational power of each head. In this paper, ablation experiments on the number of attention heads are conducted (see Section 4.2 for details). The results show that *H* = 8 achieves the optimal balance between segmentation accuracy and computational overhead; therefore, this configuration is finally adopted.

The aggregated outputs of all attention heads are concatenated, projected back to the original dimension via the projection matrix **W***_O_*, and then connected with the neighborhood mean feature $\tilde{f}$ through a residual connection. After layer normalization (LayerNorm) for stable training, the attention-aggregated feature $f_{i}^{\text{attn}}$ is obtained, as shown in Equation 3.

(3) $$f_{i}^{\text{attn}}=\text{LayerNorm}(W_{O}\cdot \text{Concat}[\sum_{j=1}^{K}\alpha_{ij}^{1}v_{j}^{1},\;\cdots ,\;\sum_{j=1}^{K}\alpha_{ij}^{H}v_{j}^{H}]+\bar{f})$$

Residual connections ensure effective gradient propagation even when attention weights degrade, and LayerNorm alleviates fluctuations in feature distribution during small-batch training.

Since cotton point clouds are derived from field measurements, they inevitably contain a small number of noisy points and samples with blurred annotation boundaries, meaning pure attention aggregation can be disturbed by outliers. To solve this problem, this study further performs adaptive fusion of the attention-aggregated features and the max-pooling features. A learnable scalar parameter *θ* is introduced to generate fusion weights via the Sigmoid function, as shown in Equation 4.

(4) $$f_{i}^{\text{out}}=\sigma (\theta )\cdot f_{i}^{\text{attn}}+(1-\sigma (\theta ))\cdot \underset{j\in N(i)}{\max}f_{j}$$

where *σ*(·) denotes the Sigmoid function and N(*i*) represents the neighborhood set of point **p***_i_*. The fusion weight σ(θ) is automatically optimized during training, allowing the model to adaptively balance the fine-grained perception capability of attention aggregation and the noise robustness of max-pooling at different levels and scales, without requiring manually fixed ratios.

#### 2.2.2 HFRG module

In the feature propagation (FP) stage of PointNet++, traditional skip connections directly concatenate shallow features with upsampled deep features, without introducing an effective feature screening and constraint mechanism. When this structure is applied to the point cloud segmentation task of cotton plants, two notable drawbacks emerge. First, the receptacle region where cotton bolls join branches is prone to semantic confusion in the shallow feature space. Unoptimized skip connections continuously propagate such confused features, which further causes segmentation deviations at structural junctions. Second, cotton branches present a slender, linear shape. Their shallow features are easily diluted and weakened by interference from surrounding background points, eventually resulting in blurred segmentation of fine branch edge contours. To tackle the above issues, this paper proposes the Hierarchical Feature Refinement Gating (HFRG) module. At each decoding level, guided by deep high-level semantic features, it performs spatially adaptive filtering on shallow skip-connection features, realizing semantics-driven fine-grained feature optimization.

In the *l*-th FP layer, the deep feature obtained by interpolation upsampling is defined as $g\in {\mathbb{R}}^{B\times C_{g}\times N}$, and the skip−connection feature output by the corresponding shallow encoder is $x\in {\mathbb{R}}^{B\times C_{x}\times N}$. Since their channel numbers usually differ, direct interactive computation is not feasible. HFRG first projects the deep feature and the skip-connection feature to a unified intermediate dimension *C*_int_ = max(*C_g_/*2,16) using two separate 1 × 1 convolutions, and stabilizes the feature distribution via batch normalization (BN), as shown in Equation 5.

(5) $$g^{\prime }=\text{BN}(W_{g}*g)\in {\mathbb{R}}^{B\times C_{\text{int}}\times N},\;x^{\prime }=\text{BN}\;(W_{x}*x)\in {\mathbb{R}}^{B\times C_{\text{int}}\times N}$$

where ∗ denotes the 1 × 1 convolution operation. Mapping the two projected feature streams into a unified feature space essentially realizes semantic calibration of shallow features using deep semantic information. The deep feature **g** completes semantic purification and high-level representation learning via multi-level set abstraction layers, and exhibits strong discriminative ability for cotton boll and branch structures. Using it as a gating guidance signal endows the generated gating weights with clear and reliable semantic foundations, overcoming the limitations of relying solely on self-judgment from shallow features.

In the unified feature space, the two projected features are added element-wise and activated nonlinearly. They are then compressed to a single channel via a 1 × 1 convolution and normalized by the Sigmoid function to generate the spatial gating map *ψ*, as shown in Equation 6.

(6) $$\psi =\sigma (W_{\psi }*\text{ReLU}(g^{\prime }+x^{\prime }))\in {\mathbb{R}}^{B\times 1\times N}$$

Each point value in the gating map *ψ* ranges within (0,1), representing the semantic confidence of the skip-connection features at the corresponding spatial position: when the value approaches 1, it indicates that the shallow features at this location are highly consistent with the deep semantic discrimination results and should be fully retained; when the value approaches 0, it represents a semantic conflict between the two, and the skip-connection features at this position need to be suppressed.

In the receptacle junction region where cotton bolls connect to branches, deep features already possess clear semantic discriminability, whereas shallow skip-connection features are prone to semantic confusion. When such conflicting features are fused, weak or even negative responses tend to occur. After being constrained by the ReLU activation, these responses produce gating weights close to 0 via the Sigmoid function, thereby blocking the upward propagation of confused features. In the main branch regions, deep semantics and shallow feature representations are highly consistent, resulting in gating weights close to 1. This fully preserves effective branch features and effectively alleviates the problem that shallow features are easily weakened and diluted by background interference.

Finally, the spatial gating map is applied to the shallow skip-connection features to obtain refined skip-connection features, which are then concatenated with the deep interpolated features and fed into the subsequent MLP to complete feature fusion at the current decoding level, as shown in Equation 7.

(7) $$\hat{x}=\psi ⊙x,\;f^{\text{out}}=\text{MLP}([\hat{x},\;g])$$

where ⊙ denotes element-wise multiplication, and the gating map *ψ* is broadcast along the channel dimension to match the dimension of feature **x**. The HFRG module is embedded in the FP2 and FP1 layers of the decoder respectively, optimizing skip-connection features layer by layer during the coarse-to-fine upsampling reconstruction process, so as to achieve hierarchical feature screening and precise correction of structural boundaries.

## 3 Materials

### 3.1 Experimental details

To ensure the fairness of comparative experiments, the reliability of results and the reproducibility of the study, all comparative models were trained from scratch without using any pre-trained weights. The experimental hardware platform is a server equipped with an NVIDIA GeForce RTX 4090 GPU (Santa Clara, California, USA), and the model code was developed based on the PyTorch v2.2.2 deep learning framework.

The two data sources, the Huaxing3Dcot dataset and the Crops3D dataset, both contain XYZ spatial coordinates and RGB color information. To eliminate the interference caused by differences in input coordinate ranges during the experiment, this study uniformly adopted XYZ coordinates combined with RGB features as the standard input for all models, and repositioned the XYZ coordinates of all point clouds with the spatial coordinate origin as the reference. The core hyperparameters in the training phase are summarized in **Table 1**. Unless otherwise specified, all datasets were strictly split into training, validation and test sets at a ratio of 7:2:1, ensuring that each model conducts objective performance evaluation under a unified data distribution.

**Table 1:** Key hyperparameters used for training all models.

Hyperparameter	Value
Optimizer	Adam
Base Learning Rate	1 × 10^−3^
Npoint	2048
Batch Size	8
Number of Epochs	300

### 3.2 Evaluation metrics

This paper employs two types of evaluation metrics, namely segmentation accuracy and model efficiency, to assess the model. For comprehensive evaluation of segmentation accuracy, mean Intersection over Union (mIoU), Overall Accuracy (OA), Precision (P) and Recall (R) are adopted as the core evaluation metrics in the experiments. The calculation formulas of these metrics are given in Equations 8–11) in turn.

(8) $$\text{mIoU}=\frac{1}{M}\sum_{i=1}^{M}\frac{{\text{TP}}_{i}}{{\text{TP}}_{i}+{\text{FP}}_{i}+{\text{FN}}_{i}}$$

(9) $$\text{OA}=\frac{\text{TP}+\text{TN}}{\text{TP}+\text{TN}+\text{FP}+\text{FN}}$$

(10) $$\text{P}=\frac{\text{TP}}{\text{TP}+\text{FP}}$$

(11) $$\text{R}=\frac{\text{TP}}{\text{TP}+\text{FN}}$$

where *M* in the formula denotes the total number of classes in the segmentation task. True Positive (TP) is the number of sample points that actually belong to the positive class and are correctly classified as positive. True Negative (TN) is the number of sample points that actually belong to the negative class and are correctly classified as negative. False Positive (FP) is the number of sample points that actually belong to the negative class but are misclassified as positive. False Negative (FN) is the number of sample points that actually belong to the positive class but are misclassified as negative.

Meanwhile, to measure the model efficiency, we report the total number of trainable parameters in the model (Params) and Giga Floating-Point Operations Per Second (GFLOPs). Params refers to the total number of trainable parameters in the model, measured in millions (M), and reflects the model’s size and memory footprint. GFLOPs measures the computational complexity of a single forward pass and serves as a key indicator of inference speed.

## 4 Results

### 4.1 Quantitative results

On the Huaxing3Dcot dataset, comparative experiments were conducted between FieldCotSeg-Net and several benchmark and state-of-the-art (SOTA) point cloud segmentation models, with the results shown in **Table 2**. The proposed model achieves an mIoU of 75.7%, an OA of 86.3%, a precision of 86.1% and a recall of 86.1%, respectively. Compared with the PointNet++ baseline model, the four metrics are improved by 4.5%, 3.1%, 3.3% and 3.0%, respectively, realizing a comprehensive enhancement of the model’s overall performance. Moreover, the increases in parameters and GFLOPs are only 0.07M and 0.1 GFLOPs, respectively, which are modest and well justified by the significant performance gains. Experimental results demonstrate that the designed ALA and HFRG modules can adaptively capture the geometric anisotropic features of different cotton organs, effectively addressing challenges such as dense leaf occlusion and significant differences in organ scales, thereby significantly improving the segmentation accuracy of cotton point clouds under complex field conditions. In addition, the segmentation accuracy of the proposed model is notably higher than that of comparative models including DGCNN and CurveNet.

**Table 2:** Performance comparison experiments with existing mainstream advanced models on the Huaxing3Dcot dataset.

Method	mIoU (%)	OA (%)	P (%)	R (%)	Params (M)	GFLOPs
PointNet	55.2	71.7	71.7	71.8	8.33	5.76
DGCNN	68.8	78.6	77.9	77.6	2.80	6.35
CurveNet	70.1	81.6	80.6	81.7	2.50	15.77
PointMLP	72.1	83.6	83.6	83.3	13.2	26.38
PointNet++ (Baseline)	71.2	83.2	82.8	83.1	1.70	4.89
FieldCotSeg-Net (Ours)	75.7	86.3	86.1	86.1	1.77	4.99

### 4.2 Ablation study

To verify the effectiveness of each core module, this study conducts a systematic ablation experiment based on the Huaxing3Dcot dataset. The experiment uses PointNet++ as the baseline network. First, we perform an ablation study on the number of multi-head attention heads in the ALA module, with the results shown in **Table 3**. Then, we construct multiple comparative structures by embedding the ALA module and the HFRG module to quantitatively analyze the influence of different component combinations on model performance. As shown in **Table 4**, both the ALA module and the HFRG module can independently improve model performance.

**Table 3:** Ablation performance of different numbers of multi-head attention heads in the ALA module on the Huaxing3Dcot dataset.

Configuration	mIoU (%)	ΔmIoU	OA (%)	P (%)	R (%)
Baseline (PointNet++)	71.2	–	83.2	82.8	83.1
Baseline + ALA Module(H = 1)	72.5	+1.3	84.6	84.4	84.2
Baseline + ALA Module(H = 2)	72.9	+1.7	84.9	84.6	84.8
Baseline + ALA Module(H = 4)	72.4	+1.2	83.6	84.5	84.6
Baseline + ALA Module(H = 8)	74.7	+3.5	85.5	85.3	85.1

**Table 4:** Ablation performance on the Huaxing3Dcot dataset.

Configuration	mIoU (%)	ΔmIoU	OA (%)	P (%)	R (%)	Params (M)
(A) Baseline (PointNet++)	71.2	–	83.2	82.8	83.1	1.70
(B) Baseline + ALA Module	74.7	+3.5	85.5	85.3	85.1	2.12
(C) Baseline + HFRG Module	73.1	+1.9	84.6	84.6	85.1	1.56
(D) FieldCotSeg-Net(B+C)	75.7	+4.5	86.3	86.1	86.1	1.77

In the ablation experiments, the number of attention heads in the ALA module was first ablated to explore the optimal number of attention heads. The choice of the number of attention heads *H* requires a trade-off between representational capacity and the feature capacity per head. Too few heads limit the geometric perception capability of each subspace, whereas too many heads result in an excessively small feature dimension per head, reducing the representational power of each head. The experimental results show that *H* = 8 achieves the optimal balance between segmentation accuracy and computational overhead. therefore, this configuration was finally adopted. Subsequently, ablation experiments were conducted on the ALA module and the HFRG module as whole modules. The experiments show that both modules independently improve model performance. Specifically, the ALA module increases the model’s mIoU by 3.5% and OA by 2.3%, while increasing the number of parameters by 0.42M. Compared to the performance gain of a 3.5% increase in mIoU, this slight increase in parameters is acceptable. The HFRG module, on the other hand, improves mIoU and OA by 1.9% and 1.4%, respectively, while reducing the number of parameters by 0.14M, effectively mitigating the parameter increase brought by the ALA module. The above experimental results effectively validate the core design concepts of this study. The ALA module can adaptively capture the anisotropic geometric features of different cotton organs via the multi-head self-attention mechanism, enhancing the model’s robustness to field variability. The HFRG module uses deep semantic features as gating criteria to spatially adaptively filter and refine the shallow skip connection features, significantly improving the segmentation boundary effect at the junction between bolls and branches, thereby laying the core foundation for the overall accuracy improvement.

The synergistic fusion of the two core modules enables the construction of the complete 3D cotton point cloud segmentation model FieldCotSeg-Net. Experimental data indicate that compared with the baseline model, the fused architecture achieves a total mIoU gain of 4.5% and a total OA gain of 3.1%. These results fully confirm that the two modules possess significant complementary and synergistic characteristics. The high-precision 3D point cloud segmentation framework built by their collaboration can fully adapt to the complex challenges of cotton point cloud segmentation and practical field application scenarios.

### 4.3 Qualitative analysis

**Figure 9:**
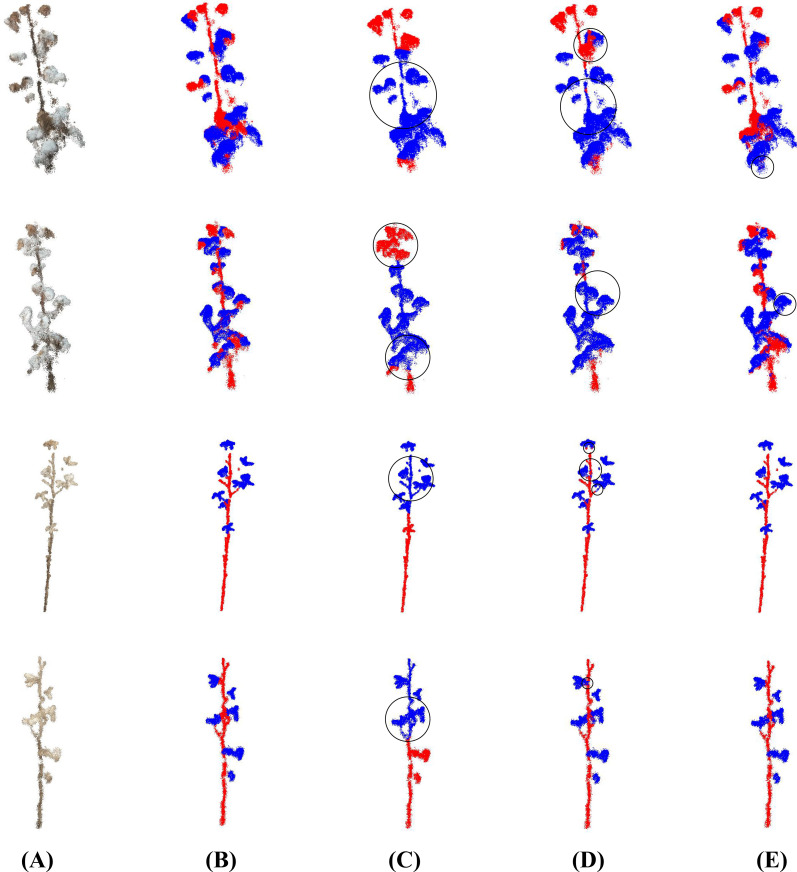
Visual comparison results of segmentation performance on the Huaxing3Dcot dataset. Compared with the PointNet model and the PointNet++ baseline model, FieldCotSeg-Net delivers more accurate segmentation results and finer detail restoration. Especially at fine structures such as crop stem-leaf junctions, it shows clearer segmentation boundaries and lower missegmentation rates. **(A)** Point Cloud. **(B)** GT. **(C)** PointNet. **(D)** PointNet++ (baseline). **(E)** FieldCotSeg-Net.

To intuitively verify the performance advantages of FieldCotSeg-Net, this study conducts a qualitative visual comparative analysis. **Figure 9** shows the segmentation results of the model on the Huaxing3Dcot dataset, and the model exhibits excellent segmentation performance for cotton samples with different morphological features. For the fine structures prone to confusion at the junctions of stems, leaves and cotton bolls, the model achieves high-precision segmentation, accurately defines organ boundaries and effectively avoids structural adhesion. Meanwhile, it can fully identify key phenotypic organs of cotton. Although the model still has a small number of segmentation deviations in local tiny regions, its overall segmentation effect is significantly improved compared with the PointNet++ baseline model. The visual analysis results support and confirm the quantitative experimental data, indicating that FieldCotSeg-Net can effectively reduce false-positive and false-negative errors in the segmentation process, which fully validates its good adaptability to point cloud segmentation tasks of cotton in complex field scenarios.

### 4.4 Generalization analysis

To fully verify the generalization performance of FieldCotSeg-Net and avoid the limitation that model performance is restricted to a single dataset, this study conducts supplementary experiments based on the public Crops3D dataset. The Crops3D dataset is acquired via terrestrial laser scanning (TLS) and contains 176 cotton point cloud samples, covering complex scenarios such as dense leaf occlusion and significant organ scale differences. These supplementary experiments systematically evaluate the cross-dataset performance of the model in cotton segmentation tasks and verify its scene transferability and task adaptability.

On the Crops3D dataset, which encompasses diverse cotton growth stages and plant morphologies, FieldCotSeg-Net still exhibits distinctly better segmentation performance than all baseline models. As listed in **Table 5**, the model obtains 74.5% mIoU and 84.1% OA, surpassing representative networks such as PointNet++ across all metrics. These results confirm the strong environmental robustness of the proposed model and its reliable adaptability to varied plant structures and complicated field backgrounds.

**Table 5:** Performance on the Crops3D cotton dataset.

Method	mIoU (%)	OA (%)	P (%)	R (%)
PointNet	57.1	70.4	70.6	70.5
DGCNN	69.6	79.3	78.3	79.9
CurveNet	71.3	81.1	80.6	80.9
PointMLP	71.6	80.2	80.9	79.6
PointNet++	71.6	81.6	81.3	82.1
FieldCotSeg-Net (Ours)	74.5	84.1	85.1	85.1

On the Crops3D dataset, which covers different cotton growth stages and plant morphologies, the segmentation performance of FieldCotSeg-Net remains significantly superior to all baseline models. As shown in **Table 5**, the model achieves a mIoU of 74.5% and an OA of 84.1%, outperforming representative networks such as PointNet++ across all metrics. Furthermore, we conducted ablation experiments on the Crops3D dataset; the experimental results are presented in **Table 6**, indicating that both the ALA module and the HFRG module effectively improve model performance even in cross-dataset scenarios. These results fully demonstrate that our model possesses strong environmental robustness and generalization ability, enabling it to reliably adapt to different plant structures and complex field backgrounds.

**Table 6:** Ablation performance on the Crops3D dataset.

Configuration	mIoU (%)	ΔmIoU	OA (%)	P (%)	R (%)
(A) Baseline (PointNet++)	71.6	–	81.6	81.3	82.1
(B) Baseline + ALA Module	73.1	+1.5	84.3	84.9	84.9
(C) Baseline + HFRG Module	72.5	+0.9	83.6	84.5	84.6
(D) FieldCotSeg-Net(B+C)	74.5	+2.9	84.1	85.1	85.1

**Figure 10:**
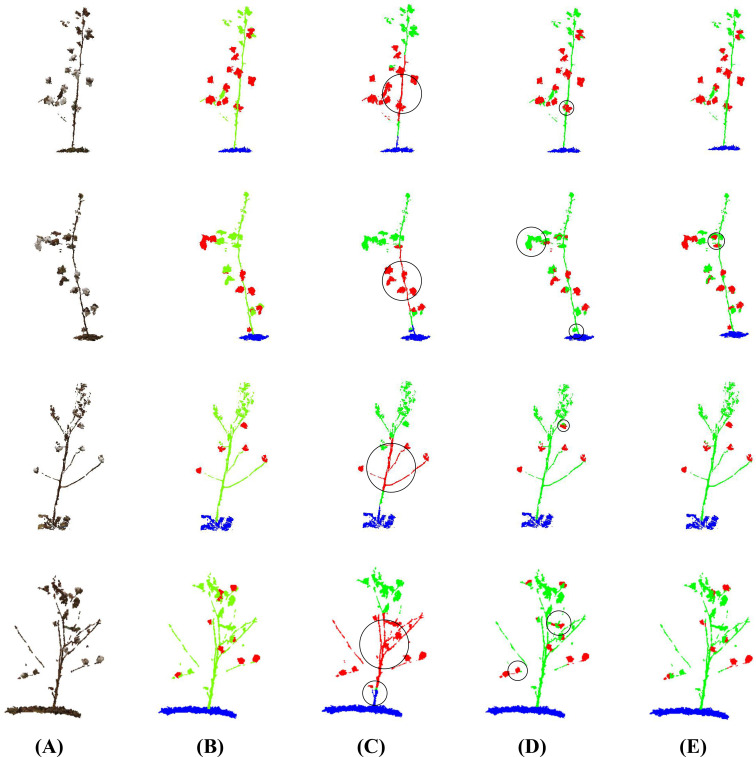
Visual comparison of segmentation results on the Crops3D cotton dataset. Compared with the PointNet model and the PointNet++ baseline model, although FieldCotSeg-Net exhibits slight segmentation deviations in a few complex scenes with dense leaf-stem occlusion or local point cloud missing, its overall segmentation accuracy and detail restoration are significantly superior to the two comparative models. **(A)** Point Cloud. **(B)** GT. **(C)** PointNet. **(D)** PointNet++. (baseline) **(E)** FieldCotSeg-Net.

The accuracy advantages demonstrated in the generalization experiments can be further supported by visual analysis. **Figure 10** shows a comparison of the segmentation effects of FieldCotSeg-Net and other point cloud segmentation models on the Crops3D dataset. By comparing the segmentation results in key regions such as the junctions of cotton bolls, stems and leaves, the refined processing capability of FieldCotSeg-Net for detailed features in complex scenes can be intuitively highlighted, further confirming that the model possesses outstanding performance superiority in cotton-specific segmentation tasks.

## 5 Discussion

### 5.1 Advantages of NeRF over SfM-MVS for reconstructing complex field-grown cotton

The conventional SfM-MVS pipeline suffers from two inherent difficulties in the 3D reconstruction of field-grown cotton canopies. First, severe occlusion exists among cotton leaves, bolls, and branches, which prevents MVS from effectively performing depth estimation in occluded regions. Second, for large-scale point cloud synthesis tasks, the time required by conventional SfM-MVS to generate high-quality point clouds is excessively long, making it impractical for real-world applications. To address these issues, this study draws on the approach of James et al. (2025). in sorghum canopy reconstruction and transfers it to the field-grown cotton scenario, selecting NeRF technology for 3D reconstruction. NeRF learns a continuous volumetric density field and a color field, and optimizes the model via differentiable rendering, thereby fundamentally bypassing the need for explicit feature matching. In the field-grown cotton scenario, NeRF exhibits two specific advantages. First, for slender branches that are partially occluded from some viewing angles, NeRF can still infer their complete geometry through consistency in transmittance from other visible angles, whereas SfM-MVS directly leaves holes at the corresponding locations due to matching failures. Second, in terms of reconstruction efficiency, NeRF can generate point clouds faster than SfM-MVS. However, there is still a gap from true real-time application, leaving substantial room for future improvement.

### 5.2 Mechanism of ALA module capturing cotton organ features

The fundamental reason why the ALA module can effectively capture slender, elongated cotton branches while distinguishing them from the boll structure lies in its directional-sensitive geometric position encoding combined with a multi-head self-attention mechanism, which achieves differentiated responses to anisotropic and isotropic structures at the feature level. For each neighboring point, the module constructs a geometric descriptor containing relative coordinates, fully preserving the spatial direction distribution of neighboring points relative to the central point. In a branch structure, neighboring points are mainly concentrated near its growth axis, while points perpendicular to the axis are sparse, exhibiting pronounced anisotropy. In contrast, a cotton boll is approximately spherical, and its neighboring points tend to be uniformly distributed in all directions, exhibiting isotropy. ALA encodes this directional information and injects it into the features, enabling the subsequent multi-head attention to adaptively adjust weights according to the direction distribution of neighboring points. For branches, the attention assigns higher weights to points located along the principal axis to capture axial continuity. For bolls, the attention weights become nearly uniform across all directions, thereby preserving their spherical geometric characteristics. This mechanism effectively overcomes the limitation of conventional single-scalar importance scores, which cannot distinguish between these two types of structural features. Furthermore, ALA adaptively fuses the attention-aggregated features with max-pooled features, balancing directional perception with robustness to field noise. It is this synergistic design of directional-sensitive encoding and differentiated response to anisotropy versus isotropy that enables the ALA module to accurately capture both slender branches and spherical bolls while reducing boundary misclassification between them.

### 5.3 HFRG’s semantically-guided gating to prevent junction misclassification

The core idea of HFRG module is to use deep semantic features as a guide to spatially adaptively filter the shallow skip-connection features, thereby blocking the upward propagation of semantically ambiguous features in junction regions. At the dense junctions between bolls and branches, shallow skip-connection features tend to confuse the boundary between spherical bolls and cylindrical branches, whereas deep features, after multiple down-sampling and semantic abstraction, have already acquired clear class discriminability. HFRG first projects the deep features and the shallow features into a unified feature space, then applies element-wise addition followed by a non-linear activation, and finally passes the result through a Sigmoid function to generate a spatial gating map. Each point in this map takes a value between 0 and 1, representing the semantic confidence of the shallow features. In the boll-branch junction region, where deep semantics and shallow features conflict, the gating values tend toward 0, effectively suppressing the continued propagation of ambiguous features. Conversely, in semantically consistent regions such as the main branch axis, the gating values tend toward 1, preserving the complete valid branch features and preventing them from being diluted by background noise. By embedding HFRG into the decoder layers FP2 and FP1 respectively, the module achieves coarse-to-fine hierarchical feature filtering, fundamentally reducing boundary misclassification between bolls and branches caused by indiscriminate skip-connection fusion.

## 6 Conclusion

This study addresses the unique challenges of organ-level semantic segmentation of field-grown cotton, namely the significant geometric differences between slender branches and spherical bolls and the severe semantic confusion in their junction regions. First, we adopt a NeRF-based point cloud acquisition and reconstruction pipeline for field-grown cotton, which effectively overcomes the geometric missing issues of conventional SfM-MVS under severe occlusion and weak-texture scenarios, providing high-quality and high-integrity point cloud inputs for subsequent segmentation. On this basis, we propose two core innovations: the ALA module and the HFRG module. The ALA module, through direction-sensitive geometric position encoding and a multi-head self-attention mechanism, achieves differentiated feature perception of the anisotropic structure of branches and the isotropic structure of bolls, effectively resolving the limitation of conventional methods that cannot distinguish the geometric characteristics of these two organ types. The HFRG module uses deep semantic features to guide the generation of a spatial gating map, adaptively filtering the shallow skip-connection features, thereby fundamentally suppressing semantic confusion and boundary misclassification in the boll-branch junction regions. Validation results on two independent datasets demonstrate that FieldCotSeg-Net, integrating the above modules, stably achieves accurate segmentation of key cotton organs at the field scale, significantly outperforming conventional methods in segmentation completeness, boundary clarity, and field robustness. This study provides a reference design paradigm for the semantic segmentation of crop organs with pronounced geometric structural differences, and is expected to promote the transformation of field-grown cotton breeding from experience-based judgment to data-driven precision decision-making, as well as to provide real-time organ location information for operational decision-making in precision agriculture.

Although preliminary success has been achieved, the reconstruction efficiency of the current framework still needs improvement, and its reconstruction stability under extreme occlusion, complex lighting, and adverse weather conditions requires further optimization. Moreover, instance segmentation at the individual cotton plant level has not yet been realized. Future research will focus on integrating temporal multiview information and weakly supervised learning strategies to extend the model’s generalization ability across different cotton varieties, growth stages, and planting densities, ultimately achieving a complete technical loop from organ segmentation to plant architectural and functional analysis, continuously serving high-throughput phenotyping of field-grown cotton and smart agriculture applications.

## Data Availability

The raw data supporting the conclusions of this article will be made available by the authors, without undue reservation.
